# An Algorithm for Sequencing by Hybridization Based on an Alternating DNA Chip

**DOI:** 10.1007/s12539-017-0220-0

**Published:** 2017-02-28

**Authors:** Marcin Radom, Piotr Formanowicz

**Affiliations:** 1 Institute of Computing Science, Poznan University of Technology, str. Piotrowo 2, 60-965 Poznań, Poland; 2 Institute of Bioorganic Chemistry, Polish Academy of Sciences, str. Z. Noskowskiego 12/14, 61-704 Poznań, Poland

**Keywords:** Sequencing by hybridization, Non-classical SBH, Algorithm

## Abstract

Sequencing by hybridization allows the reconstruction of the DNA string of a given length from smaller fragments. These fragments are obtained in the hybridization experiment in which the DNA hybridizes to a DNA chip. In a classical approach, the chip consists of all oligonucleotides of a given length, with only one type of oligonucleotide for each probe of the chip. In this paper, we propose an algorithm solving the non-classical case of SBH, where the chip probes consist set of oligonucleotides described by some specific pattern. We will present the definition of such a non-classical DNA chip and the algorithm solving a sequencing problem related to such a chip. Unlike recent metaheuristic approaches to the classical SBH problem, the proposed algorithm tries to find an exact sequence, and even in the presence of all the hybridization errors in spectrum is very often able to do so in a short time. If only negative errors from repetitions are allowed, then the algorithm is able to reconstruct sequences having length of thousands nucleotides.

## Introduction

Sequencing by hybridization (SBH) is a well-known method used for reading unknown DNA strings. It has been proposed in the late 80 s by various authors [1–3]. SBH consists of two distinct phases. It begins with the biochemical phase in which an unknown, single-stranded DNA must be prepared and cloned. In addition, the so-called DNA chip (also known as a DNA microarray, or a biochip) is being prepared. Such a chip consists of cells in which oligonucleotides are being placed. Such cells containing oligonucleotides are often called probes. Oligonucleotide is a short, single-stranded DNA fragment, usually having length within the range of 5–20 nucleotides. In the classical approach to the SBH, DNA chip is prepared, consisting of all the oligonucleotides of a given length. Four types of nucleotides are the building blocks for every oligonucleotide: adenine, cytosine, guanine, and thymine. Therefore, a string representing oligonucleotide is built over the four-letter alphabet Σ_DNA_ = {A, C, G, T}. The chip capacity depends on the length of its oligonucleotides, e.g., when the length *l* = 10, then the chip capacity *C*(10) = 10^4^ = 1,048,576 probes. Such a value is often considered as a realistic limit in the classical approach, which is strongly connected with technical difficulties in creating such a number of probes. Having prepared the cloned DNA samples and the chip, the hybridization experiment takes place. In this phase, the DNA fragments bind to the probes according to the Watson–Crick complementary rule: adenine binds to thymine, while cytosine to guanine. In other words, the DNA will attach itself to the probe if its fragment of a given length is complementary to the same length oligonucleotide in the probe. Probes with the attached DNA fragments can be detected, allowing to create a spectrum—a set of all oligonucleotides that binds with the DNA. Because one can easily transform an oligonucleotide from the probe into the corresponding fragment of the analyzed DNA molecule; in the ideal case; the spectrum consists of all fragments of a given length of the analyzed DNA. This phase is susceptible to the so-called hybridization errors: positive and negative ones. The errors of the first type occur if in spectrum, there are fragments that hybridized to the microarray when theoretically, they should not. Negative errors correspond to the loss of some fragments of the DNA in spectrum.

Obtaining a spectrum is necessary to begin the second, computational phase of the SBH. Using special algorithms, one tries to reconstruct the analyzed DNA sequence from the spectrum elements. It has been proved that when there are no hybridization errors, the computational problem which should be solved in this phase of the method is quite simple—it can be reduced to finding an Eulerian trail in the graph as stated by Pevzner in [4]. However, this assumption is unrealistic, because the SBH method is very susceptible to the hybridization errors. When the errors are present in the spectrum, the sequencing problem becomes strongly NP-hard as it has been proved in [5].

Sequencing by hybridization had been extensively modified in the last decades since its discovery, to enhance its accuracy. The method is quite sensitive to the hybridization errors in spectrum; therefore, many approaches aim to create methods that improve the resistance of the hybridization phase to errors. One of the enhancements introduced an additional information to the spectrum, i.e., estimated location in the target sequence for each of its elements [6–8]. One can here mention interactive protocols also known as sequencing in rounds [9–11]. This approach is still being improved, creating some new open questions, e.g., for the minimal number of rounds required for a given DNA to be successfully read [12]. Another way of improving SBH method is the isothermic libraries [9, 13, 14], where specially designed oligonucleotide libraries that dependent on the melting temperature are being used in the probes of the microarray. There are also recent enhancements, for example, an approach that deals with the repetitive subsequences in the target sequence [15]. In the most recent paper concerning SBH method, an approach has been presented based on the limited knowledge how many times an oligonucleotide from the probe is present in the target DNA, i.e., once or more. Knowing that simple fact can greatly improve the method, as shown in [16–18].

Various metaheuristics are also being used to further enhance SBH sequencing ability. For the already mentioned isothermic oligonucleotide libraries, a genetic algorithm has been developed [19, 20]. For the classical SBH problem, one can mention an algorithm which in its original form has been developed for the asymmetric traveling salesman problem, but has been proved to be very efficient for the SBH with both positive and negative hybridization errors [21]. In another, very recent publication authors proposed a hybrid algorithm [22] with an additional analysis concerning the relation between hybridization errors distribution and the performance of the algorithm.

Recently, Next Generation Sequencing is being extensively used, especially for genomic sequences. This approach, however, still has some drawbacks; for example, it is more suited for large DNA sequencing tasks. If the target DNA is a small sequence, using methods like SBH can still be justified. Sequencing hybridization can be used in the medical diagnostics, e.g., the [23] authors have used small libraries (8192 7-mer oligonucleotides) to inexpensively sequence individual genes or pathogen genome samples. What is even more interesting, when the reference sequence is available, sequencing by hybridization remains a reasonable approach even for the large-scale sequencing [24]. In [25], a so-called shotgun sequencing by hybridization has been proposed. Authors were able to resequence E. coli genome of 4.6 Mbp—by acquiring 3.3 million image features corresponding to 660 Mbp and 143-fold coverage. Achieved accuracy was 99.93% with 320 Mbp/day speed. All of this has been achieved using a library of 582 5-mer probes, clearly showing a potential still lying within the sequencing by hybridization methodology. In [26], authors defined mathematically three non-classical chips for the SBH hybridization phase. One of the main reasons was to reduce SBH sensitivity for the hybridization errors, especially for the so-called negative errors resulting from repetitions. They occur in spectrum when the sequenced DNA has identical fragments of a length equal to the length of oligonucleotides used in the microarray probes. Unfortunately, no algorithm dealing with non-classical spectrum resulting from such a phase has been given. The idea of such chips is based on the new type of nucleotides and in the proposed gapped and alternating chips it is the nucleotide that binds to every other classical nucleotide from Σ_DNA_ = {A, C, G, T}. The third proposed chip called binary chip uses nucleotides that bind to the specific two-elements subsets of DNA. The proposed algorithms for the gapped and the binary chips when only negative errors resulting from repetitions are present have proven to be very effective, being able to sequence unambiguously DNA sequences with length of even thousands nucleotides [27]. However, one must remember that demanding error-free hybridization experiments (except maybe for the repetitions of a sequence fragments) is in most cases impractical. SBH by its nature is susceptible to such errors; therefore, realistic algorithms for solving real sequencing problems must deal with these errors in some way. For the binary chip, an algorithm handling all types of errors had also proved to be efficient in the sequencing problem [28].

In this paper, a new algorithm is proposed for one of the non-classical variants of the SBH chips called alternating chip when all types of errors are present. The gapped chip has similar construction demands (a general-binding unspecified nucleotide), while the binary chip uses a completely different patterns for probes construction. Alternating chip is composed of probes of two different kinds, which both utilize unspecified nucleotides in their patterns. Such a nucleotide in theory must bind to any other normal nucleotides from Σ_DNA_. A resulting spectrum from a hybridization phase is divided depending on the probe type. One of these types is used to verify a path for the reconstruction of a given DNA sequence, therefore, greatly improving the chances of obtaining the original sequence. The algorithm can handle both types of hybridization errors, but can be configured to work in a mode in which only negative errors resulting from repetitions are considered. If such a spectrum can be delivered, the algorithm can reconstruct much larger DNA sequences, having even a few 1000 base pairs.

In the next section, the alternating chip will be described as proposed in [26]. Then, the hybridization errors will be discussed followed by the detailed description of the algorithm. We have tested the algorithm using different sizes of DNA sequences. In the case, where both types of hybridization errors are present, the algorithm has been tested for sequences from a range 300–700 bp. However, if only negative errors coming from sequence repetitions have been allowed within a spectrum, the algorithm has been tested with sequences ranging from 1000 up to 5000 bp. For such large sequences, the algorithm has been able to provide satisfactory results given only a minute as time limit per sequence reconstruction. The results of the computational experiments have been given followed by the conclusion with proposition of possible enhancements.

## Materials and Method

### Alternating Chip for SBH

In the paper [26], Pevzner and Lipshutz proposed three different non-classical chips for the SBH. Here, we will discuss one of them, the alternating chip. Such a microarray uses an unspecified nucleotide denoted as *x* along with normal nucleotides represented by the letters from Σ_DNA_ = {A, C, G, T}. Chip capacity tells how much probes the chip has. The total capacity of the alternating chip is ||*C*_alt_(*k*)|| = 2 × 4^*k*^. The chip is composed of all probes of two types. They are described by the following patterns:

$${{N}_{\text{1}}}x{{N}_{\text{2}}}x\ldots x{{N}_{k}}~\text{and}~{{N}_{\text{1}}}x{{N}_{\text{2}}}x\ldots ~x{{N}_{k-1}}{{N}_{k}}.$$

The number of *x* symbols is equal to *k* − 1 for the first type of probes and *k* − 2 for the second type. For both types, the number of known nucleotides denoted above as *N* is equal to *k*. These two types of probes form two sets of the hybridization spectrum. These sets are described, respectively, as *A*_1_ and *A*_2_. The length of oligonucleotides in *A*_1_ is *l*_1_ = 2 × *k* − 1, while in *A*_2,_ it is *l*_2_ = 2 × *k* − 2. While in the classical approach, each probe consists of multiple copies of exactly the same oligonucleotide, in the proposed non-classical microarray, every probe is described by some pattern. This pattern, different for each probe, defines a set of natural oligonucleotides, i.e., the ones which can be described as strings over the alphabet Σ_DNA_.

For example, in a probe denoted as C*x*G*x*G, there are 16 different types of oligonucleotides: CAGAG, CAGCG, CAGGG, ..., CTGTG. The number of oligonucleotides types in each probe is equal to 4^*k*−1^ and 4^*k*−2^ depending on the type of probe, as explained in the previous paragraph.

After the hybridization experiment, the probes which attached the analyzed DNA are used to form two subsets *A*_1_ and *A*_2_.

### Hybridization Errors

There are two types of errors that can occur during the hybridization phase: negative and positive ones. The first type is connected with a loss of information in spectrum. There are two sources of such errors. The first one is connected with the detection technology. There can be probes that did not hybridize when they should or the signal from them is so weak that it is not detected at all. There is also a second source of negative errors. The analyzed DNA can be built from two or more identical subsequences, i.e., there are repetitive fragments. Such fragments hybridize with the same probe. In our algorithm, we assume no knowledge about the number of times when different fragments hybridized with the same probe. There are articles dealing with such a problem, for example [16–18], but in our approach, we consider negative errors from repetitions as missing data.

The errors of the second type are called positive errors. They occur when for some reason probes hybridize (or are detected as such) when they should not, resulting in additional, false elements within the spectrum. When there are no hybridization errors at all, one obtains an ideal spectrum.

Depending on the type and source of errors, our proposed algorithm behaves differently. We will now discuss these different scenarios connected with the explained types of the hybridization errors.

The most simple case occurs when an ideal spectrum is obtained. The cardinality of set *S*_1_ is equal to *n* − *l*_1_ − 1, while the cardinality of set *S*_2_ equals *n* − *l*_2_ − 1. Such sets of a spectrum are subsets of *A*_1_ and *A*_2_ containing elements representing probes that hybridized to the target DNA. The length *n* of the target DNA sequence is known. It means that there are no positive or negative errors of any type within the spectrum. If that ideal case could be achieved, then the task would be to find a sequence that contains every element from both spectrum sets. Such strict conditions for the number of spectrum elements could allow quite fast and exact DNA reconstruction. The downsize of such a scenario lies in its small likelihood. In practice, hybridization errors usually occur both due to technical imperfection of the hybridization experiment and because of the repetitions of subsequences of the target DNA which cause the negative errors.

The next considered scenario assumes only negative errors within a spectrum. They can appear in both *S*_1_ and *S*_2_, which can be easily detected when the sets cardinality are less than theoretical value given in the previous paragraph. In this case, all the elements have to be used from both sets to reconstruct the DNA. This scenario is more difficult, because the algorithm must compensate the missing elements in spectrum with the ones that are present, which can lead to lowered efficiency in finding the exact DNA sequence. There is a higher probability for reconstructions which can be similar, but not exactly the same as the analyzed DNA fragment.

The third case when only positive errors are present in spectrum is not as difficult as the previous one. Having the size of the analyzed DNA and the length of oligonucleotides in the microarray, the algorithm computes the sizes of the ideal spectrum sets. Then, it only allows the solutions that contain exactly that number of elements from both sets.

The most complex case, when all types of errors are present, is unfortunately the most realistic one. The algorithm has no precise information how many elements from the spectrum have to be used. Both positive and negative errors are present and their number can only be estimated. In such a scenario, there can be many sequences given as a result of the computational phase, and without additional hybridization experiments, it is impossible to decide which one of them is the target sequence. This is a huge problem in the classical SBH approach. Algorithm we propose can handle such a realistic case, being able to produce reconstructed sequence precisely and fast.

### The Algorithm

The algorithm is able to reconstruct the analyzed DNA sequence using a spectrum obtained from a non-classical alternating chip probes in the hybridization experiment. Input data for the algorithm are as follows:

1. spectrum obtained using alternating chip, consisting of sets *S*_1_ and *S*_2_ (they are the subsets of *A*_1_ and *A*_2_ defined in alternating chip description);
2. the length *n* of the DNA fragment;
3. parameter *k* denoting the length of oligonucleotides. *l*_1_ and *l*_2_ can be computed as described in Sect. 2;
4. the sequence of the first *l*_1_ + 1 nucleotides in the analyzed DNA fragment;
5. estimated, arbitrarily taken percentage values of negative and positive errors which must exceed the real ones.

On the basis of the values of parameters *n* and *k*, the algorithm computes the theoretical number of elements that would have been needed to reconstruct the target DNA in an ideal case with no errors. Using only the elements from set *S*_1,_ a graph *G*_alt_ is constructed, in which two separate paths will be searched in commutative order—the first one for odd nucleotides of the target DNA sequence and the second one for its even nucleotides. Elements of *S*_1_ are built over the alphabet Σ_alt_ = {A, C, G, T, X}. Every element from *S*_1_ is a vertex in the graph *G*_alt_. Arcs are being created on the basis of the overlapping of letters only from the alphabet Σ_DNA_ = {A, C, G, T}. Each *S*_1_ element consists of *l*_*σ*_ = (*l*_1_ + 1)/2 letters from Σ_DNA_. Therefore, a possible overlapping is in a range from *l* − 1 to 1. Maximum overlapping of *l*_*σ*_ − 1 letters has weight equal to 1, while the minimal possible overlapping has the maximum weight equal to *l*_*σ*_ − 1. For example, vertices *AxGxG* and *GxGxC* overlap on *GxG* and *G* labels. This corresponds to two arcs going from *AxGxG* to *GxGxC*, having, respectively, weights 1 and 2.

From the input data, one has knowledge about the very beginning of the target sequence. Assuming that letter *x* in spectrum elements overlaps freely to any letter from Σ_DNA_, one can easily obtain two *S*_1_-like elements corresponding to two starting vertices in graph *G*_alt_. For example, if the starting element in the target DNA is *ACGCGAAT*, then two starting nodes (for, respectively, odd and even nucleotide paths) are: *AxGxGxA* and *CxCxAxT*. Later, we will call odd and even nucleotides paths as *P*_o_ and *P*_e,_ respectively.

**Fig. 1:**
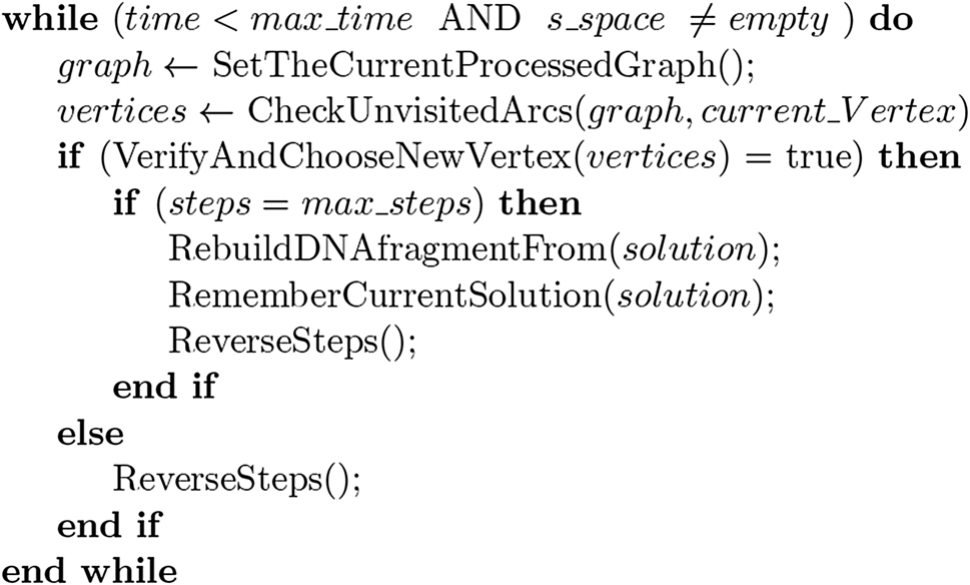
Pseudo-code for the alternating algorithm

The *x* symbol overlaps with itself and with every letter from Σ_DNA_. This feature will be used later by the algorithm. For this reason, in the phase of graph creation, overlapping only on letters from Σ_DNA_ is the only logical choice. In addition, to allow the overlapping for such a vertex labels, the normal nucleotides must be evenly placed—this is true for the elements from *S*_1_ subset, but not for the *S*_2_. For the latter, each of its elements is one base shorter than those from *S*_1_. In addition, its last two letters (from Σ_DNA_) are placed directly one after the other, without the *x* separator. The last nucleotide in each element from *S*_2_ is on the even position, like all its *x* letters. This means that if one would like to create a graph using elements from this subset, the maximal possible overlapping would be one position shorter, compared to the situation, where elements from *S*_1_ are used. This would result in a more densely connected graph, and as a result in an increased difficulty to find correct paths in it. For this reason, only elements from *S*_1_ create the search graph, while elements from *S*_2_ are being used for the verification of connections for the searched paths, as will be later explained in details. The pseudo-code for the main loop of the algorithm is in Fig. 1.

As a first step, the algorithm chooses one new vertex for the odd nucleotides path *P*_o_. Then, it chooses a new one for *P*_e_ and the procedure continues until both paths have the desired length which allows target DNA reconstruction. In each step, elements from subset *S*_2_ are being used to verify a validity of a new chosen vertex. The algorithm adds one or more nucleotides of given type (odd or even) to the reconstructed sequence depending on the overlap value of the new vertex used, i.e., a weight of a given arc taken. Weight 1 means adding a single new nucleotide, weight 2—adding two nucleotides, etc. The list of already visited vertices with values corresponding to their overlapping is denoted as solution. The vertices from both graphs are alternately put into this list. There are two important integer values steps and maxsteps. The latter represents a maximum number of vertices weights that must be reached to have odd and even paths ready to reconstruct the target sequence. The step value represents the current number of vertex weights accumulated. Every new vertex adds to steps a value equal to the weight on an arc connecting it with the previous chosen vertex in a given graph.

**Fig. 2:**
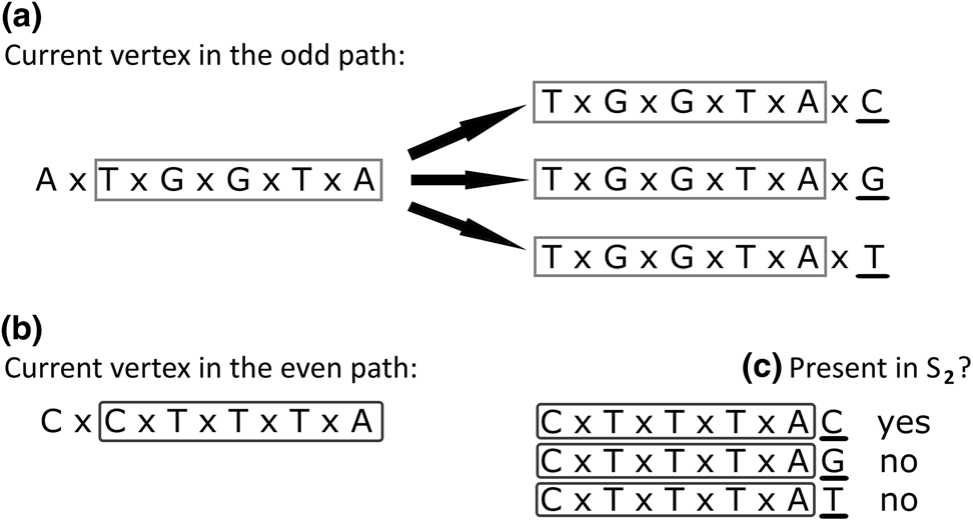
Verification of possible next move based on spectrum set S2

The verification of a new vertex is a complex process which depends on the types of errors in spectrum. The algorithm can work in four modes: no errors, only positive errors, only negative errors, or when both types of errors are present. The description of the verification process will begin with a case when there are no negative errors in *S*_2_ subset. The example of the verification process is given in Fig. 2.

In a given example, algorithm tries to extend the odd path *P*_o_. There are three possible new vertices connected by arcs with weight 1 with the vertex named *AxTxGxGxTxA* (Fig. 2a). They will add nucleotide *C, G*, or *T* (underlined black) depending on which one will be chosen. We assume that in the example, there are only vertices that have not been already visited in any of the two paths. In part b, there is a name of the last vertex already extending even path *P*_e_. Using its postfix (underlined dark grey) and last letters from vertices that can potentially extend path *P*_o,_ the verification process build potential *S*_2_-like elements (Fig. 2c). If any of them will be found in spectrum set *S*_2_, the corresponding vertex from a) part of Fig. 2 will be marked as verified. In the example, only the vertex *TxGxGxTxAxC* is verified properly. It is not possible to choose any other vertex except the ones which have been verified. Doing so would result in an incorrect reconstruction of the target DNA sequence.

**Fig. 3:**
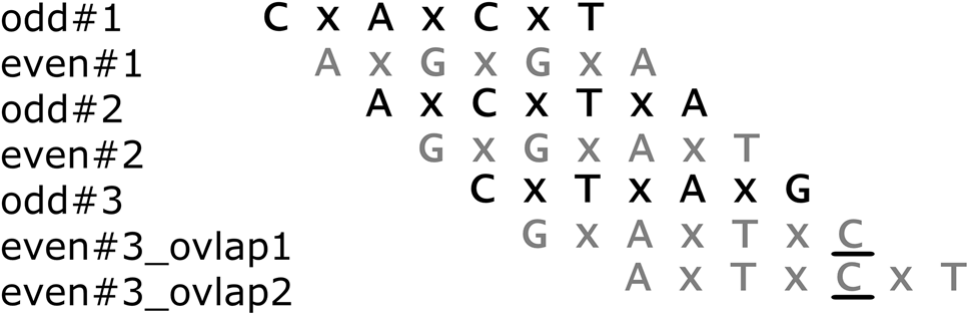
Verification with longer overlapping

As it has been stated before, vertices can also be connected with arcs having weights greater than 1. It means that they overlap on a shorter label. Such vertices, if chosen, will add two or more new nucleotides (odd or even) to the reconstructed DNA fragment. In such a case, only the first nucleotide extending a given path will (and can) be verified. An example is given in Fig. 3.

In the example, the algorithm has two possible new vertices for the even path, i.e., vertices *GxAxTxC* and *AxTxGxT*. The first one will extend the solution only by one even nucleotide *C*, and the second one will extend it by two even nucleotides *C* and *T*. They are denoted as *even#3_ovlap*1 and *even#3_ovlap*2. Verification of the first one can be performed as described in the previous example. For the second one, only the first extending nucleotide (*C*) can be verified—the procedure is analogous as for the *even#3_ovlap*1. Nucleotide *T* cannot be verified at this moment, because the algorithm is unable to create *S*_2_-like element for it. This is because vertex *odd#3* extending odd path *P*_o_ is placed there with too long overlapping (maximal in fact) to help create a proper *S*_2_ element for *even#3_ovlap*2. If the *odd#3* vertex had a shorter overlap, and therefore, it extended the odd path by more than one nucleotide from Σ_DNA_, this could be possible. As one can see, set *S*_2_ is crucial in ‘connecting’ odd and even nucleotides paths. The verification makes the search space much smaller by reducing the number of potential vertices that will extend each path. Different types of errors have different influence on the verification. Negative errors following from repetitions have no impact on the process. Even if an *S*_2_-like fragment hybridize with different parts of the DNA, it will be present in this set at least once, and the algorithm will not count how many times this element verified elements from *S*_1_. Positive errors increase the number of false verifications. The test results presented in this paper proved that their impact on the overall effectiveness of the algorithm is minimal if they are the only type of errors. The most serious situation takes place when there are negative errors in the spectrum resulting not only from repetitions but also specifically from losing data about some probes that in fact did hybridized. Elements from *S*_1_ used to create search graph can compensate for this using shorter overlapping. Unfortunately, spectrum set *S*_2_ used for verification is especially susceptible to such errors. As one can see in Fig. 3, to verify vertex *even#3_ovlap*1 element, *TxAxGC* created from postfix of *odd#3* and the last letter from *even#3_ovlap*1 must be present in *S*_2_. Its loss due to the negative errors makes such a verification impossible. Therefore, if the algorithm knows about the present of such a type of negative errors, the verification process is adjusted. Only vertices labels that overlap in *P*_o_ or *P*_e_ path on maximal possible length (i.e., *l*_1_ − 2) are verified. Shorter overlapping is accepted without verification. In our example, vertex *even#3_ovlap*1 will not be verified due to the absence of verification element *TxAxGC* in *S*_2_. However, the vertex *even#3_ovlap*2 will be accepted, containing both new letter for this path: *C* and *T*.

There is another mechanism that participates in minimizing the number of potential solutions to the sequencing problem. The paths *P*_o_ and *P*_e_ are searched in such a way that they cannot contain the same vertex from graph *G*_alt_. This comes directly from the assumption that *S*_1_ is not a multiset (neither is *S*_2_). Therefore, it is possible that a misplaced vertex in some path will be missing later in the reconstruction and will not be replaced by a vertex with longer overlapping. If the algorithm will not be able to reconstruct a sequence of a desired length, it will have to go back and try different paths. This feature makes the search process longer, but also reduces the number of ambiguous solutions. This is obviously a trade-off. If enough elements corresponding to the neighboring location in an original DNA will be missing from *S*_1_, there is a risk that the algorithm will not be able to reconstruct the correct sequence.

There are a few reasons for the algorithm to reverse the steps already taken, i.e., the vertices last taken are discarded and a new ones are being chosen. The reasons for this can be divided into two categories given in the following.

1. There are no arcs leading from a current vertex to new ones that can be chosen. In most cases, it means that there are in fact arcs, but they lead to vertices already taken or to such that cannot be verified properly.
2. The algorithm have just recently created new solution and going back is necessary to search the rest of the search space.

When the algorithm reaches the desired length for both paths, the target DNA sequence is being reconstructed. This step is simple—path *P*_o_ reconstructs all the odd nucleotides, path *P*_e_—all the even ones. If there is still time left, the algorithm reverses last steps to the last unvisited but verified vertex and try to reconstruct more sequences from this point. Ambiguous reconstructions are possible, but as the results prove such a situation is very rare. Much more likely is the situation when in a given short time, the algorithm presents unambiguous reconstruction, identical to the target DNA sequence.

When the double-path reconstruction and the verification procedures have been explained, it should be clear why this two phase approach (i.e., choosing candidates for the extension of a path, then their verification) has been implemented. It is impossible to use *S*_2_ elements for the arcs verification when the graph is being constructed. The correctness or the incorrectness of a connection between pair of vertices in a given path is based on the sequence of reconstructed nucleotides in the other path. Therefore, it is not possible to decide this before the actual sequence reconstruction begins.

As a last remark, a different approach to the algorithm construction can be considered: this time when the elements from *S*_2_ create the search graph, while set *S*_1_ is being used for the verification. Theoretically, this is possible, and one of the many differences of such an idea would be that the verification by the *S*_1_ elements would not be required to successfully connect both paths. It would of course be used to help in the reduction on ambiguous reconstructions, but the elements from *S*_2_ would suffice to connect the path, because their last two natural nucleotides are placed directly one after the other without *x* in between. Explanation why this scenario has been rejected has been in fact given in the paragraph where the search graph construction is being explained. Elements from *S*_2_ are always shorter than those from *S*_1_; therefore, the maximal possible overlapping would be shorter as well. This would create a more densely connected graph and definitely increase the search space—making the reconstruction more difficult.

## Results and Discussions

Real DNA fragments taken from the GenBank database have been used for test purposes. Various coding sequences have been merged into one, single, and long sequence. Using this long sequence, for every single test, a subsequence has been extracted by randomly selecting starting point from which the fragment of the needed length has been taken. This approach allows the necessary randomization of the test sequences, while using one very long DNA fragment from GenBank could possibly reduce the test space to one specific region of some genome. The total length of the merged sequence has been over one million base pairs to assure uniqueness of tests. Test phases are described using a few parameters, which we will now present in detail.

1. Parameter *k* describes the length of used oligonucleotides and the total capacity of the alternating chip. We used two values for *k*: 8 and 10, which correspond to 65,536 and 1,048,576 probes on a chip in each of both sets: *A*_1_ and *A*_2_. In each chip, there is the same number of probes in *A*_1_ and *A*_2_ subsets.
2. The number of errors of a given type has been prepared using a range of 0–5%. 0% has been quite difficult to obtain in the longer sequences because of the negative errors resulting from repetitions. Error preparation means adding or removing elements from the spectrum depending on the types of error for a given test phase.
3. The length *l*_DNA_ of the tested DNA fragments has been in the range between 100 and 700 bp for almost all tests except when only negative errors from repetitions have been allowed—in such a case, the DNA has been in a range from 1000 to 5000 bp.
4. Time limit for the algorithm has been set to 60 s.
5. The number of test instances for a given set of parameters 1–4 has been 100.

Negative errors from a general source have been ‘provided’ by removing the elements from the spectrum. Positive errors have been generated by taking already present elements in the spectrum and then creating its additional, slightly modified version. These new elements have been to the proper spectrum set if of course, they were not already present there. All experiments described in this section have been performed on a PC with Intel C2D processor (3.0 GHz), 8 GB RAM, and Windows 7 operating system. The algorithm has been implemented in Java.

The first test measured algorithm performance when there are no hybridization errors. In Table 1, the average number of solutions for 100 instances is given.

**Table 1:** No errors in the spectrum, average number of solutions for 100 instances for a given *k*

Capacity	DNA length						
	100	200	300	400	500	600	700
*k* = 8	1.00	1.00	1.00	1.01	1.00	1.00	1.00
*k* = 10	1.00	1.00	1.00	1.00	1.00	1.00	1.00

Clearly, one can see that this scenario is very easy for the algorithm, not only there is almost 100% chance of obtaining the precisely the target DNA sequence, but also it will be the only, unambiguous solution the algorithm gives. In the next test, we have measured the influence of only positive errors on the algorithm performance. The results are given in Table 2.

**Table 2:** Only positive errors in the spectrum, *k* = 8, average number of solutions for 100 instances

DNA length	Positive errors percent					
	0%	1%	2%	3%	4%	5%
100	1.00	1.00	1.00	1.00	1.00	1.00
300	1.00	1.01	1.00	1.00	1.00	1.00
500	1.00	1.00	1.00	1.00	1.00	1.00
700	1.00	1.00	1.00	1.00	1.00	1.00

In Table 2, the average number of solutions is given. Parameter *k* = 8 means that in both sets *A*_1_ and *A*_2,_ there are 65,536 probes. Given only 60 s for each instance, the algorithm almost always provides only one reconstructed sequence; in each case, it proved to be the hybridized target DNA. In fact, only in one case, when spectrum had 1% of positive errors and the searched DNA length was 300, in one instance (out of 100), the algorithm returned two solutions, one being the original DNA, the second a different sequence. Therefore, when for 100 instances, the number of all returned solutions had been 101, the value of the discussed cell in Table 2 is 1.01. The results in this table prove how resilient to the positive errors in the spectrum the algorithm is. Next, two Tables 3 and 4 present results for the negative errors.

As one can see in Tables 3 and 4, the smaller chip (*k* = 8) produces spectrum which can be considered inadequate for the longer sequences. On the other hand, using a chip with parameter *k* = 10 gives much greater chance for obtaining the proper reconstruction. Table 4 presents the average number of solutions given by the algorithm. For *k* = 10, a risk for ambiguous reconstruction is small even for the sequences with length of 700. In square brackets in Table 3, there is a value indicating number of instances (out of 100) when after 60 s, the algorithm has given a set of sequences, where the target DNA has been present. In parenthesis, there is a value indicating in how many instances there were ambiguous solutions (i.e., more than one sequence has been found in a given time according to the search criteria). The first value in Table 3 cells indicates the number of test instances for which the algorithm has given a non-empty set of solutions. Together with the results from Table 4, one can see that a chance for unambiguous solution with an original DNA sequence is significant even for the longer sequences. What is more important is the fact that the number of instances, where an original sequence has been found, is always very similar to the number of instances, where any solution has been found—in fact, often, these values are the same. It means that even for ambiguous solutions, there is a very high probability that the original sequence has been found and it is present in the solution set of sequences. Tables 5 and 6 present the results for both types of errors.

**Table 3:** Algorithm performance for small (*k* = *8*) and large (*k* = 1*0*) chips, only with negative errors

DNA length, *k*	Negative errors percent				
	1%	2%	3%	4%	5%
100, *k* = 8	100(99)[100]	97(97)[97]	94(94)[94]	93(93)[93]	96(92)[96]
300, *k* = 8	93(93)[93]	85(83)[84]	80(73)[79]	74(68)[72]	61(56)[60]
500, *k* = 8	67(64)[67]	54(50)[54]	44(39)[43]	24(19)[23]	11(10)[11]
700, *k* = 8	49(46)[48]	19(16) [18]	8(6)[7]	4(3)[3]	2(2)[2]
100, *k* = 10	100[99][100]	99(99)[99]	95(93)[94]	95(95)[95]	89(89)[89]
300, *k* = 10	96(95)[96]	89(89)[89]	88(86)[88]	84(84)[83]	83(83)[81]
500, *k* = 10	87(87)[87]	83(83)[83]	78(77)[78]	64(62)[64]	59(56)[59]
700, *k* = 10	87(85)[86]	75(74)[73]	56(55)[55]	50(49)[50]	47(45)[47]

Three values per table cell are as follows: the number of instances when at least one DNA sequence has been reconstructed in time, in parenthesis number of instances with only one DNA sequence obtained, in square brackets the number of instances when the target DNA has been reconstructed

**Table 4:** Only negative errors in the spectrum, average number of solutions for 100 instances for a given *k*

DNA length, *k*	Negative errors percent				
	0%	1%	2%	3%	4%
100, *k* = 8	1.01	1.00	1.00	1.00	1.04
300, *k* = 8	1.00	1.02	1.12	1.35	1.13
500, *k* = 8	1.04	1.07	1.11	3.83	1.09
700, *k* = 8	1.06	1.21	4.00	1.50	1.00
100, *k* = 10	1.01	1.00	1.02	1.00	1.00
300, *k* = 10	1.01	1.00	1.02	1.00	1.00
500, *k* = 10	1.00	1.00	1.01	1.03	1.05
700, *k* = 10	1.03	1.01	1.01	1.02	1.04

**Table 5:** Algorithm performance for small (*k* = *8*) and large (*k* = 1*0*) chips, both types of errors

DNA, *k*	Negative and positive errors percent				
	1%	2%	3%	4%	5%
100, *k* = 8	99(99)[99]	98(73)[96]	98(83)[98]	91(85)[91]	95(77)[95]
300, *k* = 8	76(67)[75]	66(45)[59]	53(36)[48]	54(36)[46]	39(29)[37]
500, *k* = 8	47(41)[46]	31(20)[28]	22(12)[19]	11(7)[7]	3(1)[3]
700, *k* = 8	31(24)[28]	18(5)[9]	4(3)[3]	3(1)[2]	3(1)[3]
100, *k* = 10	100(99)[100]	99(97)[99]	94(93)[93]	89(87)[88]	88(84)[88]
300, *k* = 10	89(84)[89]	85(81)[85]	85(76)[84]	77(70)[77]	79(75)[77]
500, *k* = 10	81(78)[80]	70(65)[69]	70(61)[69]	54(50)[53]	64(58)[63]
700, *k* = 10	76(72)[73]	67(63)[66]	46(44)[46]	45(40)[44]	47(43)[45]

Three values per table cell are as follows: the number of instances when at least one DNA sequence has been reconstructed in time, in parenthesis number of instances with only one DNA sequence obtained, in square brackets the number of instances when the target DNA has been reconstructed

**Table 6:** Average number of solutions for 100 instances, for both types of errors

DNA length	Negative errors percent				
	0%	1%	2%	3%	4%
100, *k* = 8	1.01	1.40	1.19	1.09	1.24
300, *k* = 8	1.52	1.65	2.39	2.03	1.58
500, *k* = 8	1.31	1.83	10.18	1.54	3.00
700, *k* = 8	1.29	12.44	5.25	2.00	2.00
100, *k* = 10	1.02	1.02	1.01	1.02	1.08
300, *k* = 10	1.08	1.29	1.40	1.16	1.60
500, *k* = 10	1.03	1.14	1.42	1.07	1.25
700, *k* = 10	1.06	1.05	1.04	1.22	1.21

For both types of errors, the results are similar in terms of solutions found. The main problem of such a scenario lies in an increased number of ambiguous solutions. Values in parenthesis indicating number of unambiguous solution are lower in Table 5 than in Table 3. In addition, one can see than average number of solution for such instances has increased when comparing Tables 4 and 6. As it has been explained in the section concerning the verification process, the negative errors of a general type (i.e., not only resulting from repetitions) are the main reason that the verification mechanism is not as effective as if there were no such errors in *S*_2_. One can, however, see that the number of tests where the original sequence has been present in the result set is also very similar to the number of tests where any solution has been obtained in a given short time. It means that adding positive errors to spectrum slightly worsen the performance of the proposed algorithm (especially in terms of ambiguous solutions), but it is still quite high, even for the longer sequences.

In Table 7, an interesting test is presented when only negative errors from repetitions are allowed in a spectrum. If such a situation could be achieved, the algorithm shows its potential, being able to reconstructed very long sequences exceeding a 1000 base pairs. Time limit for such a case has also been set to only 60 s. The first row tells how many times the algorithm found any solution in a given time. The second row tells about the number of solutions having the original target DNA. The third and fourth rows tell how many solutions have been unambiguous and ambiguous ones. The last one tells about the average number of negative errors resulting from repetitions in both spectrum sets *S*_1_ and *S*_2_.

**Table 7:** Only negative errors from repetitions, *k* = 10

	DNA length				
	1000	2000	3000	4000	5000
Sequence found	100/100	98/100	85/100	71/100	65/100
Target seq. found	100/100	97/100	81/100	67/100	57/100
Only 1 sequence	89/100	74/100	58/100	47/100	41/100
Many sequences	11/100	24/100	27/100	24/100	24/100
Average repetitions	26	72	108	128	177

Papers concerning DNA sequencing by hybridization sometimes offer benchmark instance sets. One of such sets has been introduced by Blazewicz et al. in [29]. In the cited paper, the set has been used to compare three metaheuristics: tabu and scatter search and two genetic algorithms enhanced GA and the proposed in the paper, the so-called revised hybrid GA. In those original tests, two threshold values for hybridization errors have been used: 5 and 20% of both positive and negative errors. What is more important and what makes the comparison of the results harder is the fact that the proposed metaheuristics can always produce a sequence that is to some degree similar to the original hybridized DNA. Therefore, a level of similarity can be compared. In our approach, the algorithm aims to reconstruct the ideal sequence, and the level of similarity can only be considered when ambiguous sequences have been found. Therefore, direct comparison of the algorithms is not possible. One can still measure for how many test sequences the algorithm can find the ideal reconstruction in a given time, and a probability that the solution in a given time will be unambiguous. We have tested a scenario when there are 10% positive and 10% negative errors within spectrum. Time limit for this case has been extended to 300 s. In Table 8, results are given. For each number of errors, there are three rows. The first row contains the information how many instances out of 40 resulted in finding the original DNA. The second one is the number of unambiguous solutions, where the third one tells how many tests have given ambiguous solutions.

**Table 8:** Proposed algorithm results for the benchmark instances by Blazewicz et al

	DNA length				
	200	300	400	500	600
Hybridization errors					
5% positive and 5% negative					
Sequence found	34/40	33/40	32/40	33/40	26/40
Only 1 sequence	34/40	33/40	31/40	33/40	26/40
Many sequences	1/40	0/40	1/40	0/40	0/40
10% positive and 10% negative					
Sequence found	25/40	21/40	24/40	19/40	12/40
Only 1 sequence	25/40	21/40	24/40	19/40	12/40
Many sequences	0/40	0/40	0/40	0/40	0/40

## Conclusion

In this paper, we have proposed a new algorithm for the DNA Sequencing by Hybridization, based on the non-classical approach, i.e., using specially designed chip called alternating. In [26], authors have given much attention to the resolving power of the proposed chips, which have been proven to be better than of the classical ones. The presented algorithm dedicated to the proposed alternating chip reconstructs DNA sequences well even when all types of errors are present in spectrum. The general simple idea behind the algorithm in this form—walking through the search space—would have been infeasible in the classical SBH because of huge number of possible reconstructions. Alternating chip offers the possibility of verification for the newly added vertices when constructing the nucleotide paths. This alone makes even such an approach possible, even more, in a very short time, the proposed algorithm can give unambiguous DNA reconstruction when both types of hybridization errors are present. Cases when more than one solution is found are rare, but as the results given in Tables 3 and 5 prove, almost always the original DNA is present in such a set of reconstructions.

Depending on the test scenario and its parameters, the results of the algorithm vary. Obviously, when longer sequences are considered with many negative and positive errors in the spectrum, the results are worse. Two important things should be considered here. The first one is the very small difference between the first and third values in Tables 3 and 5 cells. This is the difference between the cases when the algorithm gave a solution at all in a given time (the first value) and the number of times the solution was a set of sequences, where the original sequence was indeed present in a solution sets. It means that even when the algorithm gives an ambiguous solution, there is a very high chance that an original sequence is also present. In that case a smaller, low-cost hybridization experiment can be performed to identify the original DNA sequence. The second thing one should have in mind is the time the algorithm needs. The tests for only 60 s show a potential to enhance the results by just simply giving the algorithm more time.

There are many possibilities for further enhancements for the given algorithm. For example, there exist methods to reduce positive errors, based on the verification by other elements of spectrum. This is possible, however, the results proved that positive errors are not a very import problem in this approach. Another way to exploit this feature could be the modification of the hybridization experiment. One can consider a modification of the chip that makes the binding much easier (e.g., more oligonucleotides in a probe). In this way, the negative errors could probably be reduced at a cost of more positive errors (i.e., false readings). Since the latter are much more easier to handle by the algorithm, such a trade-off could result in an increase of the sequencing ability of the algorithm in a given time.

In modern SBH, various meta-heuristics are being used, like tabu search or ant colony optimization algorithms. They are the answer for the classical SBH approach, where naive search space walking is infeasible. There are no obstacles for such an approach in the non-classical Sequencing by Hybridization. Presented algorithm is an exact one, but an approach, where the sequence is constructed being similar to the target DNA is also possible. The verification feature offered by such a non-classical chip can only enhance the performance of various other algorithmic approaches aimed to improve the Sequencing by Hybridization methodology.
